# Comparison of clinical outcomes between total knee arthroplasty and unicompartmental knee arthroplasty for osteoarthritis of the knee: a retrospective analysis of preoperative and postoperative results

**DOI:** 10.1186/s13018-015-0309-2

**Published:** 2015-10-28

**Authors:** Akira Horikawa, Naohisa Miyakoshi, Yoichi Shimada, Hiroyuki Kodama

**Affiliations:** South Akita Orthopedic Clinic, Seiwakai, 96-2 Kaidoushita, Showa-Ookubo, Katagami, 018-1401 Japan; Department of Orthopedic Surgery, Akita University Graduate School of Medicine, 1-1-1 Hondo, Akita, 010-8543 Japan

**Keywords:** Clinical outcome, TKA, UKA

## Abstract

**Background:**

Excellent results have recently been reported for both total knee arthroplasty (TKA) and unicompartmental knee arthroplasty (UKA), but there have been few reports about which has a better long-term outcome. The preoperative and postoperative results of TKA and UKA for osteoarthritis of the knee were thus compared.

**Methods:**

The results of 48 patients who underwent TKA and 25 patients who underwent UKA were evaluated based on clinical scores and survivorship in the middle long-term period. Preoperative, latest postoperative, and changes in the femoro-tibial angle (FTA), range of motion (ROM), Japanese Orthopedic Association score (JOA score), and Japanese Knee Osteoarthritis Measure (JKOM) were compared. The patients’ mean age was 73 years. The mean follow-up period was 9 years (TKA: mean, 10.5 years; range, 7–12 years; UKA: mean, 9 years; range, 6–11 years).

**Results:**

Preoperative FTA and ROM were significantly higher in the UKA group than in the TKA group. Total changes in all scores were similar among the two groups, as were changes in scores for all JOA and JKOM domains. The cumulative revision rate was higher for UKA than for TKA (7 versus 4 %). Kaplan-Meier survivorship at 10 years was 84 % for UKA and 92 % for TKA.

**Conclusions:**

This clinical study found no significant differences between TKA and UKA, except in long-term survivorship.

## Background

Total knee arthroplasty (TKA) and unicompartmental knee arthroplasty (UKA) have been recognized as good choices for the treatment of progressive osteoarthritis of the knee since these surgical procedures were first invented and reported [1, 2]. TKA has long been acknowledged as the best operative treatment for knee arthritis due to its durability and effectiveness in the recovery of knee function [3–6]. UKA has recently been established as a minimally invasive approach that preserves the bone and has excellent range of motion (ROM), less blood loss, and easier recovery of muscle damage [5]. Although UKA is reported to have poorer durability than TKA, there are many reports about its excellent clinical outcomes, which are not inferior to those of TKA.

To the best of our knowledge, few clinical studies and review articles have compared the long-term survivorship and clinical outcomes after TKA and UKA [7].

## Methods

Patients who underwent knee joint arthroplasty between 2004 and 2007 with either a TKA (Stryker Scorpio NRG, Japan Stryker Company, Tokyo, Japan) or fixed-bearing UKA (Stryker EIUS UKA) were retrospectively identified from our database and reviewed. There were 48 patients with 50 primary TKAs and 25 patients with 28 UKAs performed at our institution. This study was performed in accordance with the ethical standards established in the 1964 Declaration of Helsinki and its amendments, and was approved by the local ethics committee. The exclusion criteria for UKA were more than 15° varus deformity, over 5° flexion contracture, less than 90° active ROM, dysfunction of the anterior cruciate ligament (ACL), non-isolated medial compartment involving the patellofemoral joint, or rheumatoid arthritis [7–10]. Those who met any of the exclusion criteria for UKA underwent TKA. All patients with follow-up clinical data were enrolled in this trial, and the data were collected prospectively. The clinical analysis data included preoperative and postoperative femoro-tibial angle (FTA), ROM, Japanese Orthopedic Association (JOA) scores [11], and Japanese Knee Osteoarthritis Measure (JKOM) [12]. The mean follow-up period was 9 years (TKA: mean, 10.5 years; range, 7–12 years; UKA: mean, 9 years; range, 6–11 years). Survivorship was defined as freedom from revision surgery. All TKAs were performed by the medial parapatellar approach, which induced eversion of the patella [13]. The UKA surgical procedure was a mini-invasive technique that involved a medial parapatellar approach with a 1-Qfb (Querfingerbreite, about 1.5 cm) incision from the upper pole of the patella to 1-Qfb distal to the medial tibial plateau by subluxation of the patella and exposure of the ACL [14]. All surgeries were performed by a single surgeon (H.K.). After the operation, patients were encouraged to undergo physiotherapy with mobilization and weight-bearing under the assistance and control of a physiotherapist. Clinical outcomes, such as FTA, ROM, and JOA scores, were assessed at 2 weeks, 1 month, 3 months, 6 months, 1 year, and the latest follow-up. The clinical outcomes were then compared between the TKA and UKA groups. At every follow-up, a clinical and radiographic review was performed. Differences in sex distribution were assessed using the chi-square test. Differences in BMI, age, and follow-up time between TKA and UKA were evaluated using the chi-square test or non-matched pair analysis for two-group comparisons. All outcome measures (FTA, ROM, JOA score, JKOM) were evaluated preoperatively and postoperatively by the Mann-Whitney *U* test.

Kaplan-Meier survival analysis was performed to assess implant durability. Statistical analysis was performed using Microsoft Office Excel and Statcel 3 (OMS, Inc., Tokyo, Japan).

## Results

**Fig. 1:**
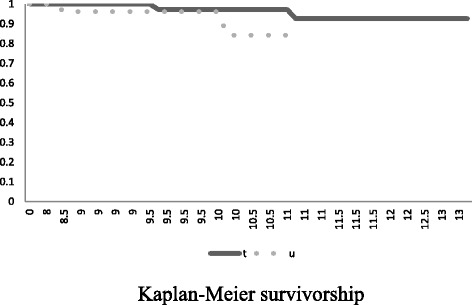
Kaplan-Meier survivorship comparison at 10 years between TKA and UKA. A significant difference exists in survivorship between TKA and UKA. t: TKA. u: UKA

The mean age was not significantly different between the TKA group (72.2 ±7.9 years) and the UKA group (74 ± 6.4 years). The BMI was also not significantly different between the TKA group (25 ± 4.0 kg/m^2^) and the UKA group (24.1 ± 3.6 kg/m^2^) (Table 1). Preoperative and postoperative FTA and ROM were higher in the UKA group than in the TKA group. There were no differences between the preoperative and postoperative JOA scores and JKOM (Tables 2 and 3). The cumulative revision rate was higher for UKAs (7 %) than for TKAs (4 %; *p* = 0.469). The cause of revision was tibial implant sinking or infection (Table 4). Kaplan-Meier survivorship at 10 years was 92 % for TKA and 84 % for UKA (Fig. 1).

**Table 1:** Comparisons of variables in the TKA and UKA groups

	TKA group (*n* = 50)	UKA group (*n* = 28)	*p* value
Age (years)	72.2 ± 7.9	74 ± 6.4	0.060^a^
Sex (% female)	80	90	0.550^a^
Height (cm)	153 ± 4.2	150 ± 5.3	0.015^b^
Weight (kg)	54.8 ± 8.9	50.2 ± 7.6	0.023^b^
BMI (kg/m^2^)	25 ± 4.0	24.1 ± 3.6	0.201^b^
Follow-up period (years)	9	10.6	0.055^a^
Indication (primary osteoarthritis)	40	20	0.607 ^a^
Revision (number)	2	2	0.469^c^

^a^χ^2^ test

^b^Student’s *t* test

^c^Mantel-Haenszel procedure

**Table 2:** Preoperative FTA, ROM, and JOA scores

	TKA group (*n* = 50)	UKA group (*n* = 28)	*p* value
FTA	189.8 ± 7.9	180 ± 6.4	0.001
ROM	12.3–126.7	8.3–142	0.150
JOA	60.5 ± 4.2	66.5 ± 5.3	0.148
JKOM	50.1 ± 8.9	58.3 ± 7.6	0.238

Mann-Whitney *U* test

**Table 3:** Postoperative FTA, ROM, and JOA scores

	TKA group (*n* = 50)	UKA group (*n* = 28)	*p* value
FTA	175 ± 7.9	172.1 ± 6.4	0.200
ROM	3.3–126	4.2–142.5	0.015
JOA	81.1 ± 4.2	80.2 ± 5.3	0.98
JKOM	78.3 ± 10.4	83.6 ± 9.2	0.186

Mann-Whitney *U* test

**Table 4:** Cases that required revision arthroplasty

	TKA	UKA
Number	2	2
FTA (average)	175	170
Cause of revision		
Infection	2	
Aseptic loosening		2

## Discussion

Recent studies have identified changes in scores as a measure of the effects of TKA and UKA [15, 16], and in this study, the focus was improvement in function with these procedures. The present study confirmed previous reports that UKA had significantly better postoperative outcome measures (FTA and ROM). These patients, however, also had higher preoperative scores, and it is the change in scores that determines the effect of the intervention. These results might be ascribed to the differences in knee joint contracture, osteoarthritic changes, and surgical invasion between the two groups. The changes in scores for all JOA and JKOM domains demonstrated no differences between the groups. If we consider the changes in scores, both surgical procedures are equally effective in improving function. However, it is important to recognize that there is a ceiling effect to scoring systems currently in use when evaluating patients with knee arthroplasty postoperatively [7].

Survival analysis is an accepted method of evaluating the durability of prostheses, and the prostheses showed survival rates of 92 % for TKA and 84 % for UKA. Although TKA is more frequently performed due to this perception, it is a more durable operation. Therefore, we assumed that clinical outcome scores for TKA and UKA would show similar excellent changes from preoperatively to postoperatively, and TKA would demonstrate better survivorship than UKA.

There were some limitations to this study. First, UKA and TKA cases were unevenly distributed, with the number of UKA cases being only about 60 % than that of TKA cases. It is reasonable to assume that surgeon proficiency could affect the outcome of UKA; however, surgeon-specific differences in outcomes were not identified. In our view, the present patient cohorts reflect the relative UKA/TKA usage in the general population undergoing knee arthroplasty.

Next, the ages of both patient groups were relatively older compared to other reports. Although some studies suggested that UKA had better absolute postoperative clinical outcomes, the average age of patients in those studies was within 70 years [17, 18]. In addition, the patients in those studies also had higher preoperative scores, which might imply high physical activity levels. In other words, the present patients had relatively low physical performance due to their age, and this may have affected the results for recovery of clinical outcome.

Finally, UKA demonstrated a higher failure rate but tended to have non-inferior function. About 10 % of the present UKA cases were revised, compared with 4 % for TKA. Early survival studies of UKA demonstrated revision rates of 15 to 28 % and midterm survivorship of 84 % to 98 %[19–21], while TKA has established survivorship of 92 to 100 % in long-term studies [1, 4–6, 16, 22–25]. As far as the present study is concerned, revision surgery was required due to sinking of the tibial implant, which might have been affected by overconcentration of loading because of overcorrection to an FTA of 170°. It has been suggested that surgeons should avoid cutting tibial bone stock too much and avoid overcorrection under 170° valgus because of the function of the ACL [26, 27]. Loss of tibial bone stock will lead to fragility and inability to sustain mechanical stress loading, and overcorrection of valgus might lead to ACL dysfunction as it is recognized as the stabilizer of knee alignment. In the present cases that required revision surgery, there may have been overcorrection (about 170°), which might have affected the function of the ACL and promoted overloading of the tibial implant, leading to its collapse.

In summary, consistent with the literature, patients with UKA had non-inferior clinical outcome scores for function preoperatively and postoperatively compared to patients with TKA. Changes in clinical outcome scores were similar among the two groups. The durability of both prostheses was assessed by survival analysis and showed that TKA was more durable.

## Conclusion

There were no significant differences in outcomes between TKA and UKA, except for long-term survivorship, in the present study. This may suggest that both surgical procedures provide excellent results if we select patients carefully based on their age, activities, knee function, and degree of osteoarthritis.
